# Effects of a midwife psycho-education intervention to reduce childbirth fear on women’s birth outcomes and postpartum psychological wellbeing

**DOI:** 10.1186/s12884-015-0721-y

**Published:** 2015-10-30

**Authors:** Jennifer Fenwick, Jocelyn Toohill, Jenny Gamble, Debra K. Creedy, Anne Buist, Erika Turkstra, Anne Sneddon, Paul A. Scuffham, Elsa L. Ryding

**Affiliations:** Menzies Health Institute Queensland, Griffith University, University Drive, Meadowbrook, QLD 4131 Australia; Gold Coast University Hospital, Parklands Drive, Southport, QLD, 4215 Australia; University of Melbourne, Grattan Street, Parkville, Victoria 3010 Australia; Karolinska Institutet, SE-171 77 Stockholm, Sweden

**Keywords:** Childbirth fear, Midwife psycho-education, Caesarean section, BELIEF study

## Abstract

**Background:**

High levels of childbirth fear impact birth preparation, obstetric outcomes and emotional wellbeing for around one in five women living in developed countries. Higher rates of obstetric intervention and caesarean section (CS) are experienced in fearful women. The efficacy of interventions to reduce childbirth fear is unclear, with no previous randomised controlled trials reporting birth outcomes or postnatal psychological wellbeing following a midwife led intervention.

**Method:**

Between May 2012 and June 2013 women in their second trimester of pregnancy were recruited. Women with a fear score ≥ 66 on the Wijma Delivery Expectancy / Experience Questionnaire (W-DEQ) were randomised to receive telephone psycho-education by a midwife, or usual maternity care. A two armed non-blinded parallel (1:1) multi-site randomised controlled trial with participants allocated in blocks of ten and stratified by hospital site and parity using an electronic centralised computer service. The outcomes of the RCT on obstetric outcomes, maternal psychological well-being, parenting confidence, birth satisfaction, and future birth preference were analysed by intention to treat and reported here.

**Results:**

1410 women were screened for high childbirth fear (W-DEQ ≥66). Three hundred and thirty-nine (*n* = 339) women were randomised (intervention *n* = 170; controls *n* = 169). One hundred and eighty-four women (54 %) returned data for final analysis at 6 weeks postpartum (intervention *n* = 91; controls *n* = 93).

Compared to controls the intervention group had a clinically meaningful but not statistically significant reduction in overall caesarean section (34 % vs 42 %, *p* = 0.27) and emergency CS rates (18 % vs 25 %, *p* = 0.23). Fewer women in the intervention group preferred caesarean section for a future pregnancy (18 % vs 30 %, *p* = 0.04). All other obstetric variables remained similar. There were no differences in postnatal depression symptoms scores, parenting confidence, or satisfaction with maternity care between groups, but a lower incidence of flashbacks about their birth in the intervention group compared to controls (14 % vs 26 %, *p* = 0.05). Postnatally women who received psycho-education reported that the ‘decision aid’ helped reduce their fear (53 % vs 37 %, *p* = 0.02).

**Conclusion:**

Following a brief antenatal midwife-led psycho-education intervention for childbirth fear women were less likely to experience distressing flashbacks of birth and preferred a normal birth in a future pregnancy. A reduction in overall CS rates was also found. Psycho-education for fearful women has clinical benefits for the current birth and expectations of future pregnancies.

**Trial registration:**

Australian New Zealand Controlled Trials Registry ACTRN12612000526875, 17th May 2012

## Background

Few randomised controlled trials (RCT) have tested interventions to reduce childbirth fear in pregnant women and improve birth outcomes and emotional wellbeing. The first of two previous Finnish RCTs reported improved vaginal birth rates in fearful women who had either six cognitive behavioural sessions or two intensive sessions with an obstetrician [1]. In the second study women who received six group psycho-education sessions with a psychologist had lower CS rates compared to women in the control group. [2]. Obstetrician counselling or group psycho-education with a psychologist enhanced preparedness for birth and positive parenting in women with intense fear [1, 3]. However no published RCTs have used the skills of midwives.

The BELIEF (Birth Emotions and Looking to Improve Expectant Fear) trial investigated the efficacy of a midwife-led psycho – educational intervention for reducing women’s fear during pregnancy. The protocol for the study has been published [4]. Antenatal outcomes of the RCT showed a reduction in pre-birth fear level (W-DEQ), improved childbirth self-efficacy (CBSEI), and a trend to reducing decisional conflict (DCS) and depressive symptoms (EPDS) [5]. This current paper reports secondary outcomes for the RCT at 6 weeks postpartum with respect to mental health and obstetric outcomes of women receiving the intervention compared to controls.

### Childbirth fear

Childbirth fear has been recognised and investigated in Scandinavian countries for more than three decades [6]. In Sweden women are routinely treated for childbirth fear within multidisciplinary teams but managed predominantly by midwives [7, 8]. In Australia, childbirth fear has only recently started to attract greater attention [9]. Toohill et al. [10] reported high fear, as measured by the W-DEQ (score ≥66), to affect approximately 20 % of Australian childbearing population. This figure is similar to others reported by Swedish, Canadian and United Kingdom (UK) researchers over the last 15 years [10]. However more recently there has been a focus on identifying severe levels of fear (W-DEQ ≥85). This level of fear appears to occur in about 10 % of women, with some indication that it may be slightly higher in European countries, and is said to impact women’s daily functioning (such as attending work or mothering the baby) [11, 12].

A woman’s emotional and psychological wellbeing contributes significantly to her perceptions and experiences of pregnancy and birth. Poor emotional health is associated with increased childbirth fear and risk of depression [13], birth trauma [14–17], an inability to interact positively with the baby and meet infant developmental needs [18, 19], and can be a stressor to the couple relationship [19, 20]. In addition, pregnant women with childbirth fear more often prefer a caesarean section (CS) [12, 21]. They are also at increased risk of obstetric interventions such as elective or emergency CS [22]. In the absence of routine screening and intervention, fearful Australian women may be at higher risk for CS than their northern European counterparts who receive education and support.

High CS rates are of concern across industrialised countries due to higher physical and psychological morbidity [23], the impact to women’s reproductive life as a result of a scarred uterus [23] and the high likelihood of undergoing a repeat CS [24]. The cost of a CS is at least twice that of a non-operative birth in low risk women [25]. The influence of fear on women’s birth decisions and how operative birth adds to the pervasiveness of fear in populations is of international interest [26–34].

### Objectives

As part of our BELIEF RCT we hypothesised that women receiving midwife-led telephone psycho-education during pregnancy would report improved postnatal mental health six weeks after birth, experience higher levels of vaginal birth (reduced CS) and prefer a vaginal birth in a subsequent pregnancy compared to the control group.

## Method

A two armed non-blinded parallel (1:1) multi-site randomised controlled trial was used. Details of the RCT have been published previously [4, 5]. To summarise, women between 12 to 24 weeks gestation, aged 16 years and older, able to read, write and understand English and with capacity to consent were invited to participate. Women who required an interpreter, or had a fetal diagnosis of major abnormality or incompatibility with life were excluded. Women were recruited by research midwives in antenatal clinics of three metropolitan teaching hospitals in south-east Queensland, Australia between May 2012 and June 2013. Participants provided their written consent to the study. Human research ethics approval was obtained from Griffith University and Queensland Health multi-site hospital Human Research Ethics Committee for the three participating hospitals.

### Data collection and measures

Immediately following consent to participate, women were asked to complete a questionnaire that sought data about demographic characteristics, obstetric history and psycho-social factors. The W-DEQ, which has been validated within the Australian context [9], was used to measure antenatal childbirth fear [35]. Women scoring high childbirth fear (≥66) were randomised to the BELIEF intervention or control group. An evidenced based birth decision aid booklet was provided to all randomised women. The booklet included information about common practices used around the time of birth, health outcome statistics associated with interventions and tools for decision-making and communicating consumer needs with care providers.

Further questionnaires were completed at 36 weeks gestation to ascertain course of pregnancy, and at six weeks postpartum to determine birth outcomes. At both time-points depression was measured using the Edinburgh Postnatal Depression Scale (EPDS) [36]. The ten item EPDS has shown both high sensitivity and specificity when used in the antenatal and postnatal periods [37]. At 6 weeks postpartum women completed the self-efficacy subscale of the Sense of Confidence and Satisfaction Scale which measures the extent to which a mother is confident in her abilities to effectively nurture her child [38]. In addition women were asked ‘*How often have you experienced distressing ‘flash-backs’ to your labour and birth since having your baby?*’ For more details see the study protocol [4].

Women who had not returned questionnaires at six weeks after birth were telephoned to prompt completion of questionnaires. These could be completed over the telephone or by hard copy returned by free post. After two reminders by telephone including calling alternative numbers or sms, and after an additional questionnaire was mailed with no response, women were considered lost to follow-up.

### Intervention

Women in the BELIEF intervention group received psycho-education sessions at 24 and 34 weeks gestation by telephone at a scheduled time convenient to them. Psycho-education sessions were around 1 hour duration (First session range: 22–125 min; Second session range: 10–104 min) [5]. Women randomised to the control group received usual maternity care at their chosen facility.

The BELIEF study aimed to review women’s current expectations and feelings around fear of childbirth, support the expression of feelings, and provide a framework for women to identify and work through distressing elements of childbirth [4, 5]. A detailed description of the intervention has been published [4].

### Outcomes

The secondary outcomes of the BELIEF study reported in this paper tested the efficacy of the intervention in reducing caesarean section, induction of labour (amniotomy, prostaglandin or syntocinon), epidural use in labour and neonatal admission to special care or intensive care nursery. Psycho-social outcomes included lower levels of depressive symptoms (EPDS), distressing flashbacks of the birth and improved parenting confidence (PSOC). Women’s satisfaction with their ultimate birth mode and the decision aid assisting with decreasing feelings of fear are also reported.

### Sample size

The sample was calculated after allowing for 30 % attrition, using a significance level of 5 %, power of 80 %, and a two tailed test. A sample of 150 women in each group was determined to detect a 10 point reduction in high fear scores between the intervention and control groups pre-birth for the primary outcome.

### Randomisation

A research assistant not involved in recruitment or provision of the intervention accessed the randomisation service following receipt of participant’s written consent and completed baseline measures. Participants were allocated in blocks of ten and stratified by hospital site and parity using a centralised web-based service to either intervention or control group. A midwife providing the intervention was subsequently notified of women’s details to initiate contact and the intervention.

### Statistical methods

SPSS Version 21 [39] was used for all analyses. Descriptive statistics were generated for all demographic variables and scale scores. Chi square tests were used to compare groups on categorical outcome variables and independent t-tests were conducted to compare continuous scale scores. An alpha level of 0.05 was used for all statistical tests. The Cronbach alpha values, which indicate internal consistency reliability, for the scales administered at Time 3 were .88 EPDS and .90 for the seven items of the self-efficacy and satisfaction sub-scale of the Sense of Confidence and Satisfaction Scale.

## Results

**Fig. 1:**
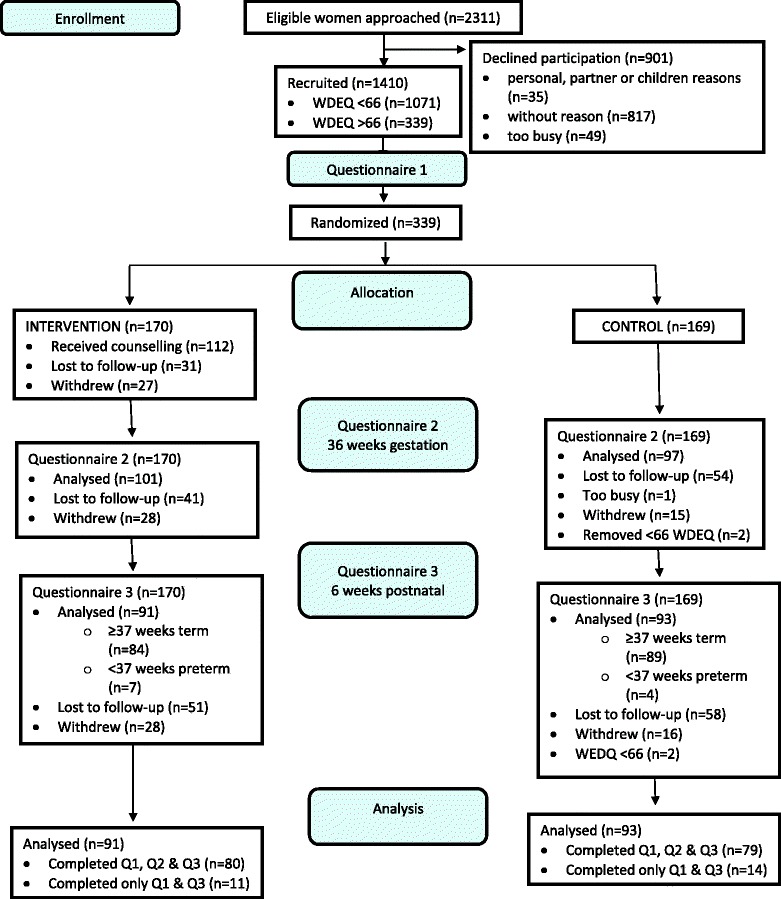
Study Flow Diagram

Of 1410 women recruited into the study 339 (24 %) reported high childbirth fear and were randomised to their allocated study groups. One hundred and eighty-four (54.2 %) women provided data at completion of the study within the allocated time frame (10 weeks). Two women were incorrectly randomised and removed from analysis. (Refer Fig. 1 Study flow diagram).

Participants characteristics were representative of Australian women giving birth [10]. Psycho-social characteristics of the baseline population have been reported elsewhere [40].

Preliminary analyses were conducted to compare those women who completed both the baseline and postpartum questionnaires, and those that were lost to follow-up. There was no difference in the proportion of women in the intervention group and the control groups that dropped out of the study (46.5 % and 45 % respectively, *p* = .78). There was also no difference between the completed and lost to follow up groups on parity (*p* = .94), marital status (*p* = .50), preferred mode of birth (*p* = .29), baseline WDEQ scores (*p* = .35) or baseline EPDS scores (*p* = .79). The women in the lost to follow-up group were however younger (*p* = .006), less educated (*p* < .001), with lower household incomes (*p* < .001).

### Mode of birth and obstetric events

No statistical difference in birth mode was found between the intervention and control groups. However fewer women in the intervention group had a CS (34 % vs. 42 %, *p* = 0.27). In particular, there was a lower emergency CS rate in the intervention group (18 % vs. 25 %, *p* = 0.23) (Table 1). Women having their first baby in the intervention group had higher rates of vaginal birth (65 % vs. 55 %) (See Table 2). At 6 weeks postpartum fewer women who received psycho-education preferred a CS for their next pregnancy (18 % vs 30 %, *p* = 0.04). Refer to Table 1. There were no differences for induction of labour, epidural use, neonatal admission to a nursery or satisfaction with birth mode.

**Table 1:** Outcomes six weeks post-partum

Variable	Intervention group (*n* = 91)	Control group (*n* = 93 )	*P*	*CI*
Prefer CS next birth, n (%)	16 (17.6)	28 (30.1)	0.04	0.24 – 0.99
SVD, n (%)	44 (48.4)	39 (41.9)	0.38	0.72 – 2.31
Forceps/ vacuum, n (%)	16 (17.6)	15 (16.1)	0.79	0.51 – 2.40
CS, n (%)	31 (34.1)	39 (41.9)	0.27	0.39 – 1.30
Elective CS, n (%)	15 (16.5)	16 (17.2)	0.88	0.43 – 2.05
Emergency CS, n (%)	16 (17.6)	23 (24.7)	0.23	0.31 – 1.32
Induction of labour, n (%)	34 (37.4)	27 (29.1)	0.25	0.76 – 2.75
Missing	11 (12.1)	13 (14)		
Narcotic in labour, n (%)	26 (28.6)	29 (31.2)	0.65	0.44 – 1.66
Missing	11 (12.1)	12 (12.9)		
Epidural analgesia, n (%)	33 (36.3)	33 (35.5)	1.00	0.53 – 1.94
Missing	13 (14.3)	13 (14)		
Preterm birth, n (%), range weeks	7 (7.7), 32–36	3 (3.2), 28–35	-	-
Admit to nursery, n (%)	16 (17.6)	18 (19.4)	0.75	0.42 – 1.87
Mother readmission, n (%)	3 (3.3)	5 (5.4)	-	-
Baby readmission, n (%)	8 (8.8)	6 (6.5)	-	-
B/fed 6weeks P/N, n (%)	76 (83.5)	73 (78.5)	0.38	0.66 – 2.91
Flashbacks of birth, n (%)	13 (14.3)	24 (25.8)	0.05	0.22 – 1.01
EPDS Mean (SD), range	6.2 (5), 0–22	5.5 (4.7), 0–23	0.30	-.67 – 2.14
Satisfaction birth mode	53 (58.2)	61 (65.6)	0.30	.40 – 1.32
Satisfaction of Decision Aid in Decreasing fear, n (%)	48 (53.3)	34 (37)	0.02	-.31 - -.02
Parenting confidence Mean (SD), range	32.4 (6.2), 10–42	33.3 (6.6), 15–42	0.32	−2.81 – 0.94

*CS* caesarean section, *SVD* spontaneous vaginal delivery, *B/fed* breastfed, *EPDS* Edinburgh Postnatal Depression Scale, *CI* confidence interval 95 %

**Table 2:** Birth mode by parity

	Vaginal birth		Emergency caesarean		Elective caesarean	
Study Group	Nullip n (%)	Multip n (%)	Nullip n (%)	Multip n (%)	Nullip n (%)	Multip n (%)
Intervention	33/51 (64.7)	27/40 (67.5)	15/51 (29.4)	1/40 (2.5)	3/51 (5.9)	12/40 (30.0)
Control	29/53 (54.7)	25/40 (62.5)	19/53 (35.9)	4 /40 (10.0)	5 /53 (9.4)	11/40 (27.5)

### Psychological factors

There were no differences between the groups for depressive symptoms (EPDS: Mean 6.2 vs. 5.5 *p* = 0.30) or parenting confidence (Mean 32.4 vs. 33.3 *p* = 0.32). Fewer women in the intervention group, however, reported having flashbacks (*p* = 0.05). Women in the intervention group reported they gained more from the decision aid booklet compared to women in the control group (*p* = 0.02).

## Discussion

The 8 % lower rate of CS in women who received midwife psycho-education is a noteworthy finding. While not statistically significant, this result is in line with the findings of Saisto and colleagues [1] who similarly demonstrated that CS rates could be reduced through antenatal counselling with fearful women. However while Saisto et al. [1] included women seeking elective CS of any parity, they did exclude women not eligible for a vaginal birth. Consistent with the work of Rouhe et al. [2] we also found vaginal birth rates were improved for women having their first baby. Once again, however, eligible criteria differed with Rouhe et al. [2] excluding women with significant psychological problems and only randomising women with severe levels of fear (W-DEQ >100). As our primary outcome was to reduce women’s antenatal fear levels regardless of birth mode our sample included women who had previously experienced a CS, women carrying a multiple pregnancy and women with other mental health disorders. Consequently our study sample included women at higher obstetric and possibly higher psychological risk than the previous two trials, and included women with lower fear levels (WDEQ-A ≥ 66). While we were optimistic, it was unlikely that we would reduce elective CS rates given it is difficult for women in Australia to secure vaginal birth following a previous CS. The lower overall CS rate in the intervention group (34 % vs. 42 %) aligns with the Queensland [41] state average, indicating that CS rates in fearful women can be reduced to at least those of the general population. In our study this can be attributed most particularly to reducing emergency CS, however both emergency and elective CS was lower for primiparous women who received the intervention. We noted CS rates could have been further improved had clinical practice been similar at each study site given women in the intervention group at one site had half the CS rate to that of women in the control group.

Similar to previous studies [1, 2] there were no differences between groups for use of pharmacological analgesia, induction of labour or neonatal outcomes. Comparisons to work undertaken in Finland [2] shows that our Australian cohort of fearful women reported using epidural anesthetic during labour around half as often as women in Finland. This may be due to lower vaginal birth rates in our RCT and is also consistent with higher CS trends in Australia [41]. However induction of labour was more frequent in our Australian study population (33 %) compared to Finnish women (20 %) [2].

Randomised controlled trials to date indicate that women’s anxiety [1] and/or fear levels [2, 5] are improved following antenatal counseling and this translates to higher satisfaction with vaginal birth. We did not specifically determine women’s satisfaction with their birth experience, but women were satisfied with the type of birth they eventually had. The statistically significant difference in women’s lower preference for caesarean section in their next pregnancy could perhaps indicate that the midwife intervention assisted women to feel positive about normal childbirth in both the short and longer term. Perhaps this also explains why women in the intervention group were less likely to experience distressing flashbacks of their birth despite no differences found in postpartum depressive symptoms or parenting confidence.

Flashbacks may be a symptom of trauma. Women who received the intervention may be in a more positive psychological frame of mind about childbirth. We have previously reported that women receiving the intervention had higher levels of confidence and a trend to lower decisional conflict during pregnancy [5]. Postnatally, women in the intervention group also assessed the decision aid more positively than the control group. Women in the intervention group were encouraged to challenge care when they were uncomfortable or uncertain. This may increase women’s sense of being active within the decision making process and thus emotionally protective. This is important given the strong link between birth trauma symptoms, fear, and requests for CS; and could have future health care savings in relation to potentially reducing CS rates.

### Limitations

There are a number of limitations to consider when interpreting the results of this secondary analysis. While sample size was powered to detect a difference in the primary outcome of childbirth fear, this difference should have also been sufficient to show statistical differences in overall CS rates based on a previous study [1]. Inclusion in the current study of women who were not eligible for vaginal birth may have impacted our findings for birth mode. In addition, despite using a number of engagement strategies our postnatal attrition rate was much higher than expected. Younger, less educated women with lower household incomes were harder to engage. Future studies that include all risk pregnancies need to recruit larger samples of women and incorporate additional strategies to minimise attrition in vulnerable childbearing women. The sample included women attending publicly-funded antenatal clinics. The proportion of women who give birth in private hospitals in Australia is 29 % [41]. Consequently, findings cannot be generalized to women with childbirth fear who receive private care. Additionally study sites adopted different practices in respect of women’s labour and birth care, for example; considerably higher CS rates were shown at two sites compared to the third site. The reasons for this were not investigated in this study and therefore unable to be extrapolated to wider birthing communities.

## Conclusion

This is the first RCT to report midwife counseling for women with childbirth fear. Women who received the psycho-education had lower rates of CS compared to controls, and this was associated to lower rates of emergency CS. Additionally women reported fewer flashbacks of their birth indicating that midwife counseling for fear may also reduce the development of trauma symptoms. These are important findings in relation to improving normal birth rates and women’s emotional wellbeing. The study supports the role of midwives in assisting women with high levels of childbirth fear.
